# Structured Breathing Exercises as a Mental Health Intervention for Young Individuals with Anxiety and Depression: A Randomized Controlled Trial Protocol

**DOI:** 10.1192/j.eurpsy.2026.11687

**Published:** 2026-07-31

**Authors:** P. Hjorth, A. Bicik

**Affiliations:** Institute of Regional Health Research, Odense, Denmark

## Abstract

**Introduction:**

Young people with anxiety and depression frequently experience physiological hyperarousal, emotional dysregulation, and impaired stress tolerance. Standard treatments, while effective, are limited by side effects, accessibility, and adherence challenges. Structured breathing exercises (SBE) represent a promising complementary approach. By promoting parasympathetic activation and reducing stress-related arousal, SBE may improve emotional stability and mental well-being.

**Objectives:**

This protocol outlines a planned randomized controlled trial (RCT) designed to evaluate the efficacy of SBE in reducing anxiety and depressive symptoms in young individuals aged 16–24 years. Secondary objectives include assessment of general well-being and functional outcomes.

**Methods:**

The trial is planned to commence in early 2026, pending funding approval. We aim to recruit 100 participants from a youth mental health support program in Denmark. Participants will be randomized to an intervention group receiving SBE or a control group. The intervention comprises a four-week program including diaphragmatic breathing, box breathing, and Tummo breathing, practiced daily via a mobile application and supported by weekly group sessions. Primary outcomes will be measured with the Hospital Anxiety and Depression Scale (HADS), while secondary outcomes include the General Health Questionnaire (GHQ-28). Assessments will take place at baseline, post-intervention, and follow-up.

**Results:**

We hypothesise that SBE will reduce symptoms of anxiety and depression by enhancing autonomic regulation, improving stress resilience, and strengthening emotional self-regulation. Participants are expected to report improved concentration, energy levels, and well-being.

**Conclusions:**

This protocol describes a scalable, non-pharmacological intervention targeting mental health in young people. If funded and successfully conducted, the trial will provide important evidence for integrating structured breathing techniques into psychiatric care and youth mental health programmes.

**Disclosure of Interest:**

None Declared

